# Supplementary material for: Functional IKK/NF-κB signaling in pancreatic stellate cells is essential to prevent autoimmune pancreatitis
Source: Commun Biol. 2022 May 27;5:509. doi: 10.1038/s42003-022-03371-3 (PMC9142538; doi:10.1038/s42003-022-03371-3)
Supplement: Supplementary file 2 — Supplementary Information [file 42003_2022_3371_MOESM2_ESM.pdf]

Supplementary Figures

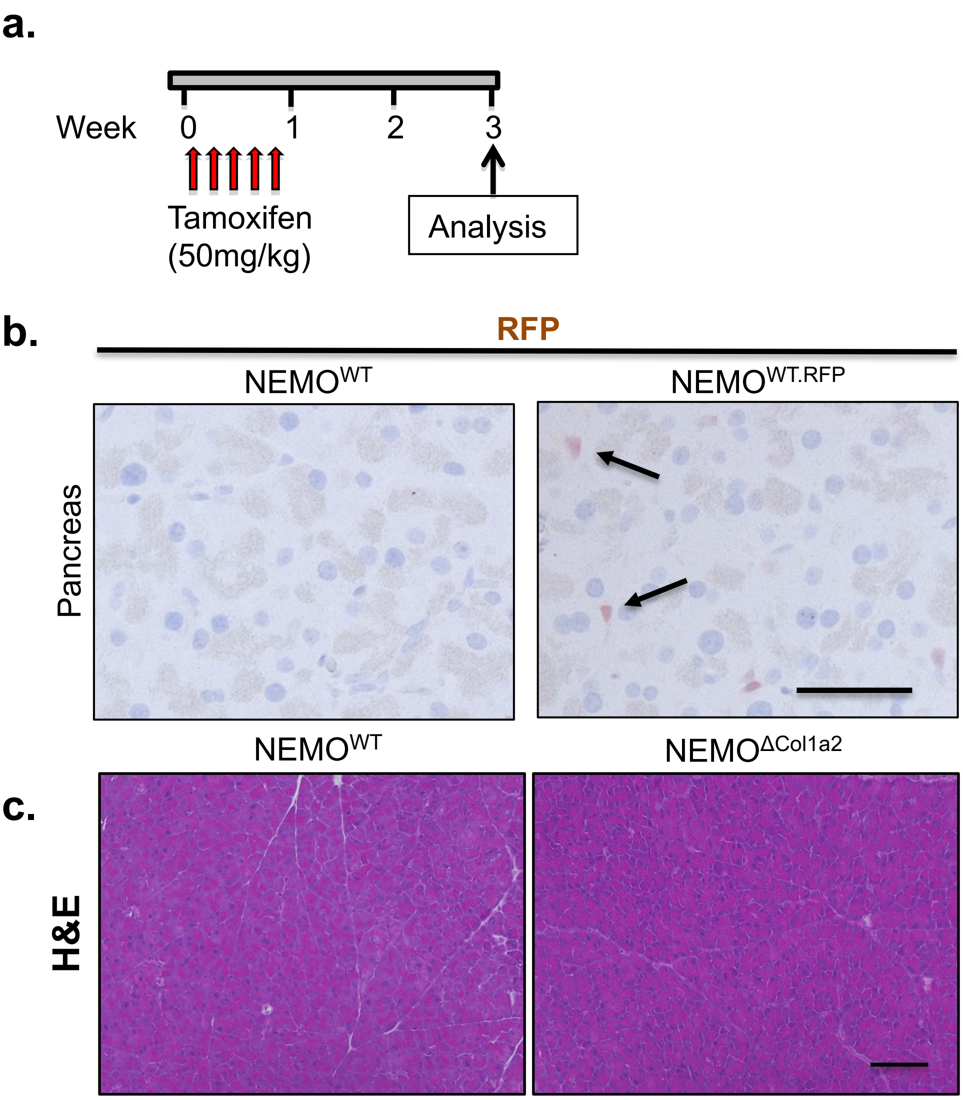

Supplementary figure 1. Visualization of Cre recombinase activities in the pancreas. a) Scheme of tamoxifen injection. b) RFP staining showing positive cells (arrow) in the pancreas. Scale bar: 50µm. c) Histology of pancreata 3 weeks after the starting of the first tamoxifen injection. Scale bar: 100µm.

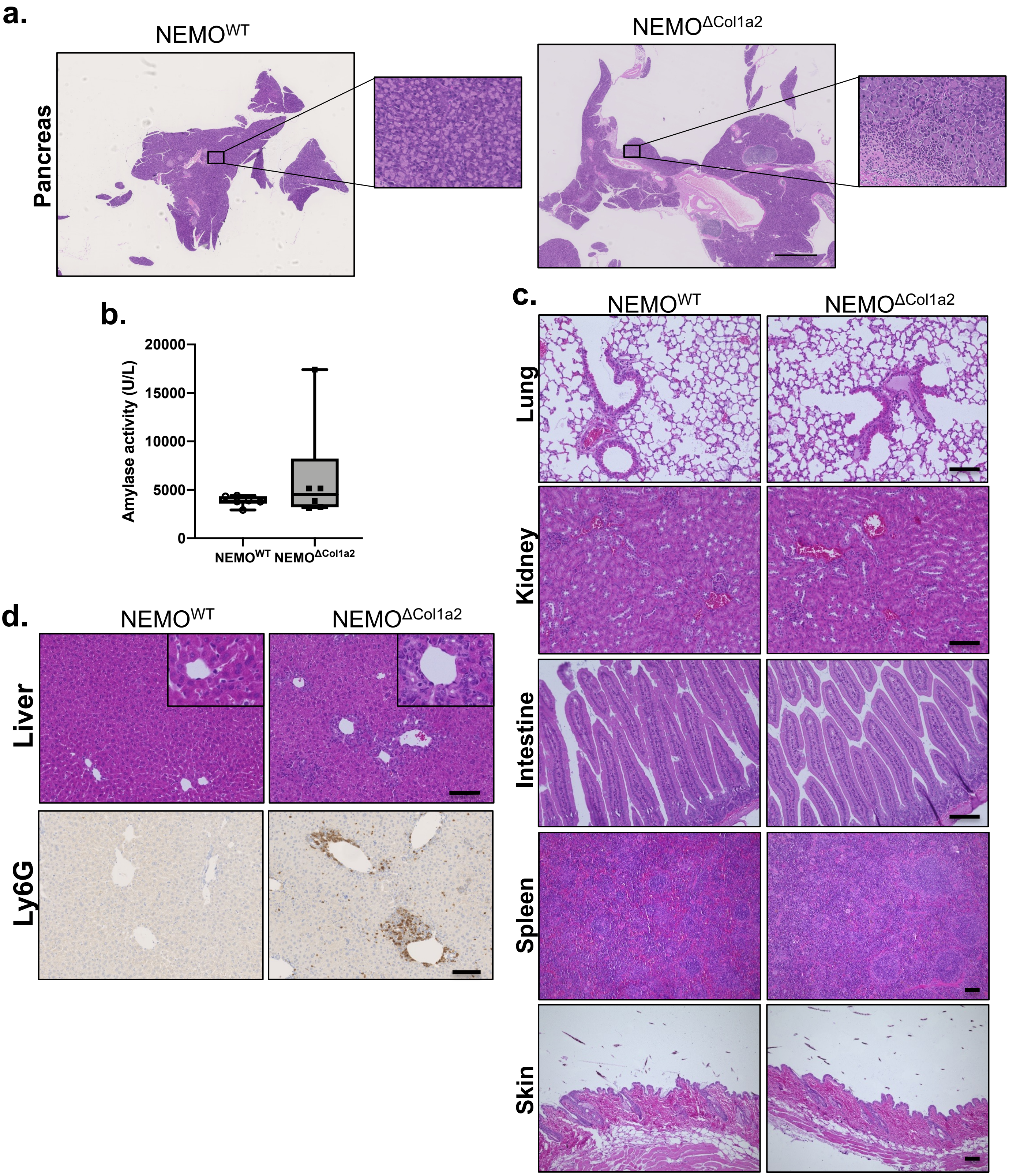

Supplementary figure 2. Long-term tamoxifen diet induces spontaneous pancreatitis in NEMO$^{\Delta Col1a2}$ animals. a) H&E staining of pancreas. Scale bar: 1 mm. b) Amylase activities measured from serum (n=6). c) H&E staining of different organs. Scale bar: 100µm. d) Periportal inflammation in the liver of NEMO$^{\Delta Col1a2}$ mice. Scale bar: 100µm. The n number represents biological replicates.

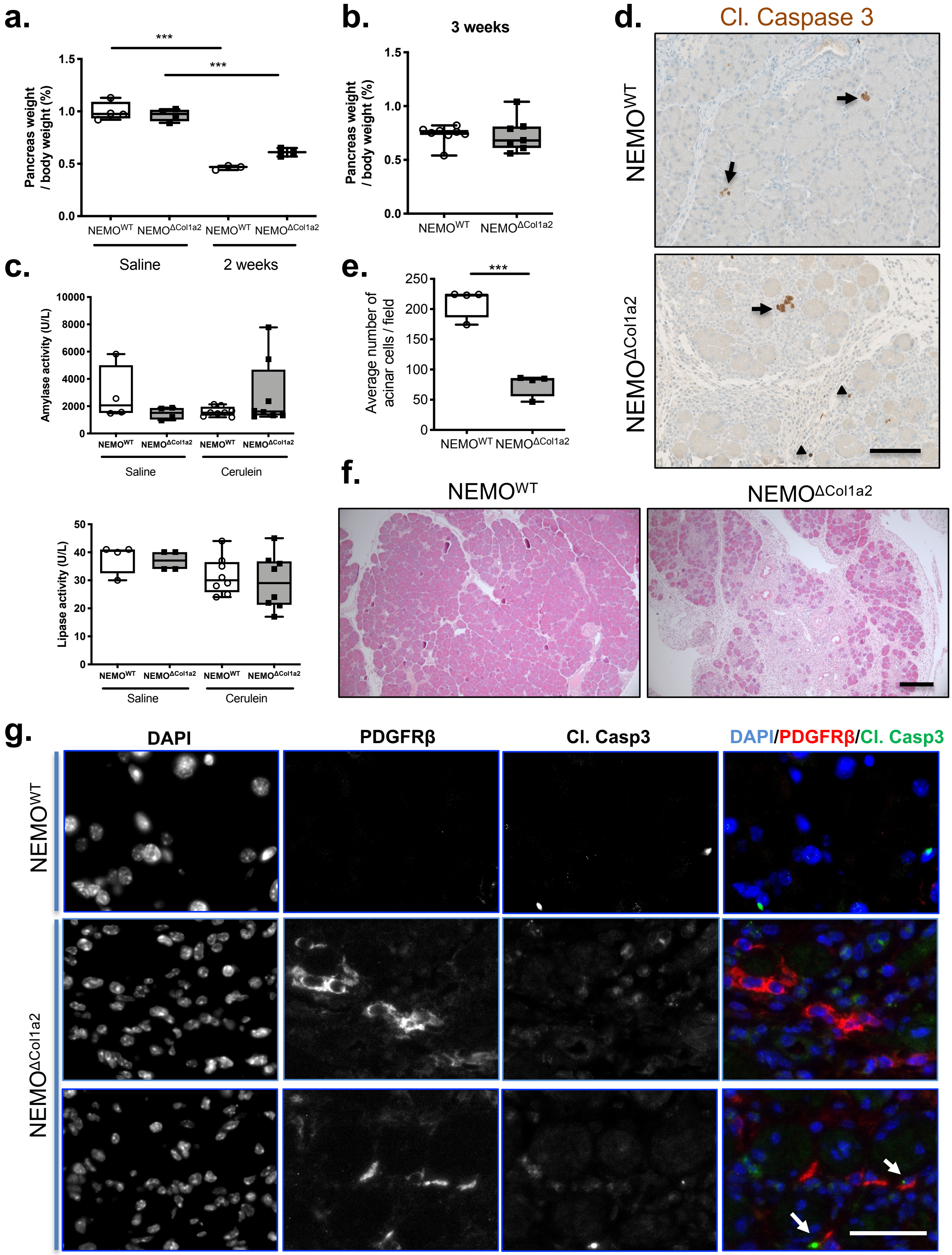

Supplementary figure 3. Characterization of animals after caerulein and tamoxifen injections. a) Measurement of the pancreas weight as a ratio of the body weight from the saline- and caerulein-injected animals (n≥3). b) Ratio of pancreas weight to the body weight from animals at 3 weeks (n=8). c) Amylase and lipase activities measured from serum (n≥4). d) Cleaved caspase 3 staining indicating the presence of apoptotic cells. Arrow: apoptotic acinar cells; arrow head: apoptotic non-parenchemal cells. Scale bar: 100µm. e) Quantification of the number of acinar cells per field on pancreas sections (3 week) (n=4). f) Low magnification of pancreas histology. Scale bar: 200µm. g) Co-staining of PDGFRβ and cleaved caspase 3 to identify apopotic PSCs. Arrow: apoptotic PSCs. Scale bar: 25 µm. Whiskers: Min to Max. T-test (two-tailed): ***p<0.001. All n numbers represent biological replicates.

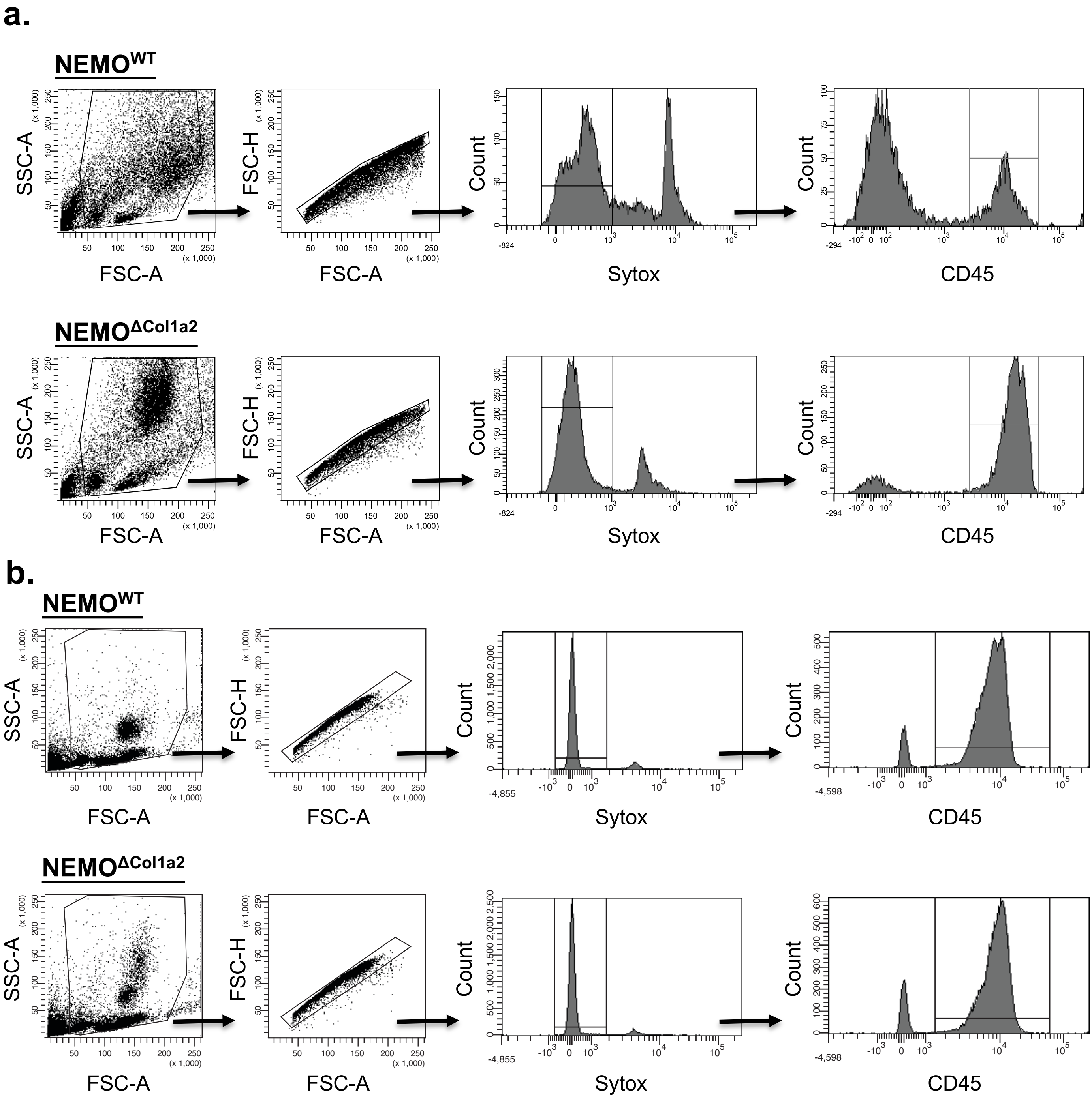

Supplementary figure 4. FACS analyses on the infiltrating cells from pancreata and immune cells in the peripheral circulation. Cells were isolated from pancreata (a) using gradient centrifugation or from blood (b) of $NEMO^{WT}$ and $NEMO^{\Delta Col1a2}$ animals .

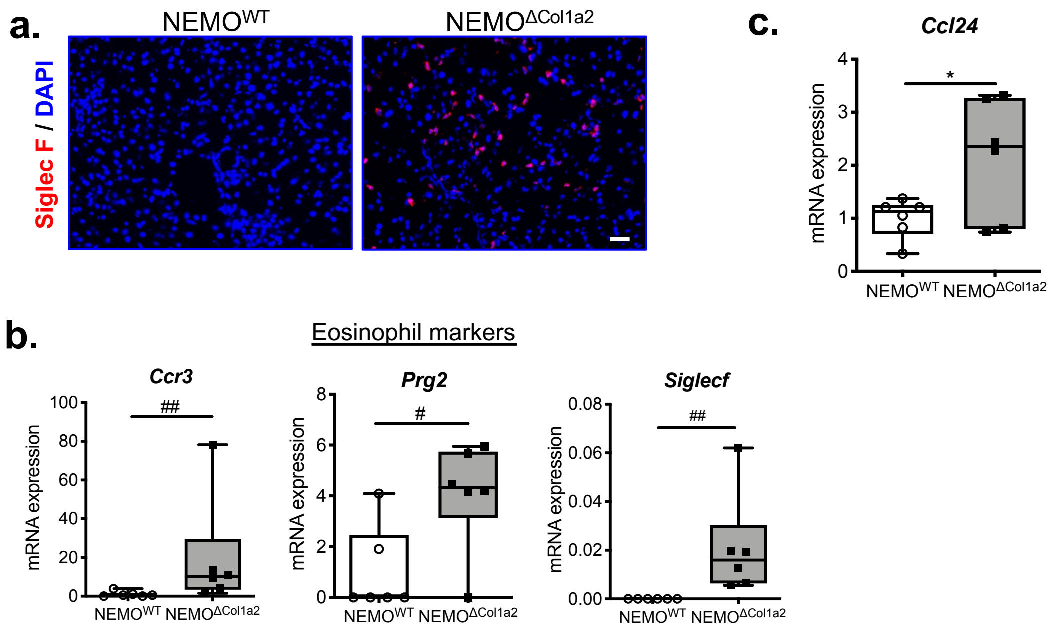

Supplementary figure 5. Eosinophils are recruited to the pancreas of NEMO$^{\Delta Col1a2}$ mice after long-term tamoxifen diet. a) Siglec-F staining showing eosinophil infiltration in the pancreas. DAPI: nuclei. Scale bar: 50µm. b-c) Expression of the chemokine CCL24 and eosinophil markers (CCR3, Siglec-F and PRG2) from pancreatic tissue assessed by qPCR (n=6). Whiskers: Min to Max. Mann-Whitney (two-tailed): #p<0.05; ##p<0.01. T-test (two-tailed): *p<0.05. The n number represents biological replicates.

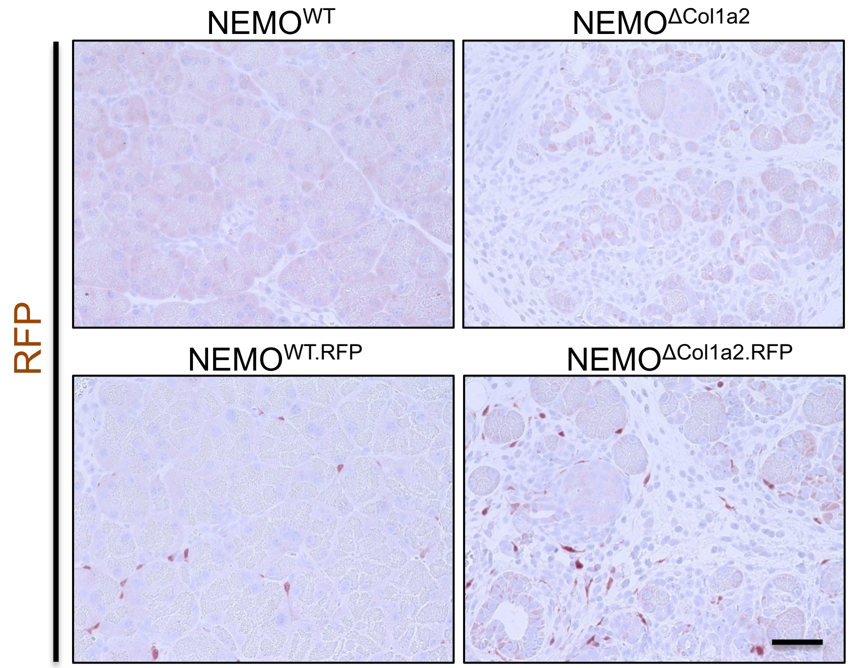

Supplementary figure 6. Analysis of the RFP reporter mouse lines. RFP stainings on the $NEMO^{WT}$, $NEMO^{\Delta Col1a2}$, $NEMO^{WT.RFP}$ and $NEMO^{\Delta Col1a2.RFP}$ pancreata 3 weeks after caerulein and tamoxifen injections. Scale bar: 50µm.

a.

GSEA in NEMO$^{\Delta Col1a2}$ animals (vs. NEMO^{WT})

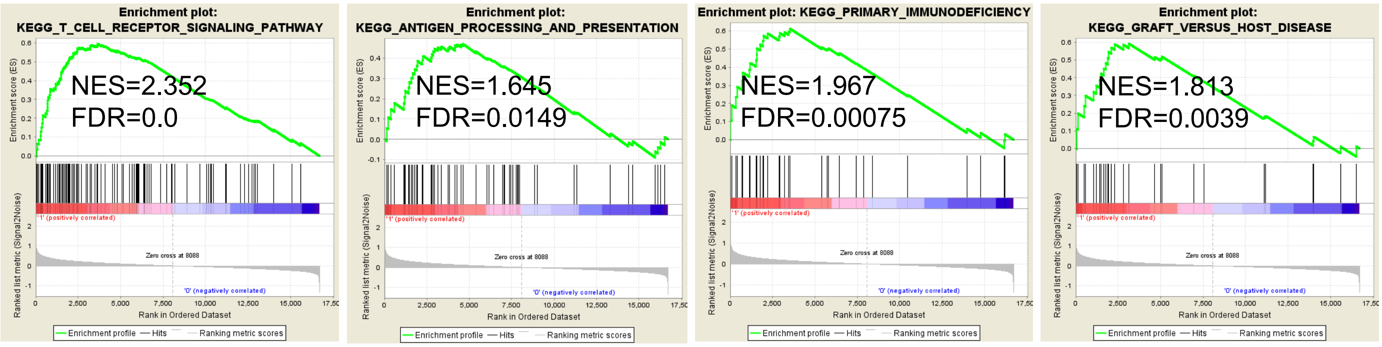

b.

GSEA in human AIP (vs. alcoholic CP)

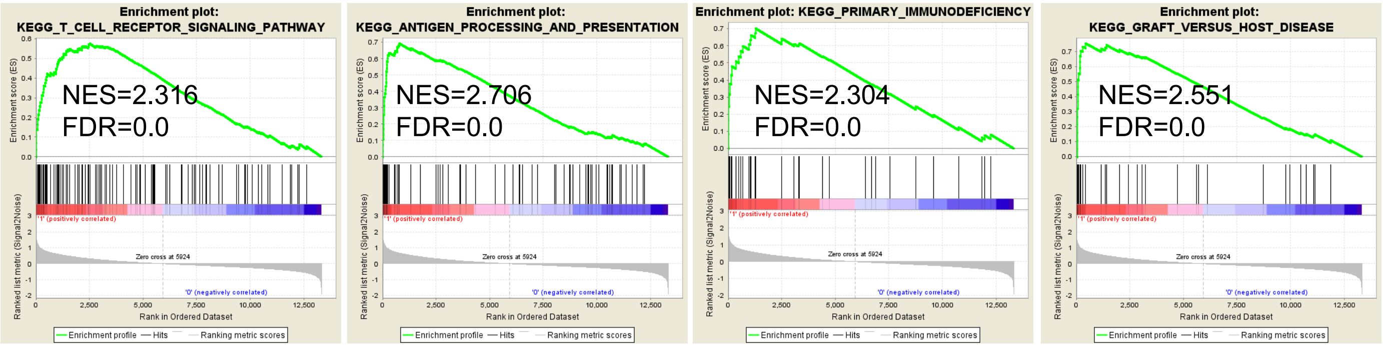

Supplementary figure 7. Comparison of the gene expression profiles between NEMO$^{\Delta Col1a2}$ mice and human AIP samples. DNA microarray performed on the pancreatic samples of NEMO^{WT} and NEMO$^{\Delta Col1a2}$ animals collected at the 3-week time point. a) GSEA was performed on the microarray results of the NEMO$^{\Delta Col1a2}$ group with the NEMO^{WT} group as a reference (n=3). b) Analysis of microarray data from AIP patients (n=4) using GSEA with AICP patients (n=5) as a reference. NES: Net enrichment score. FDR: False discovery rate.

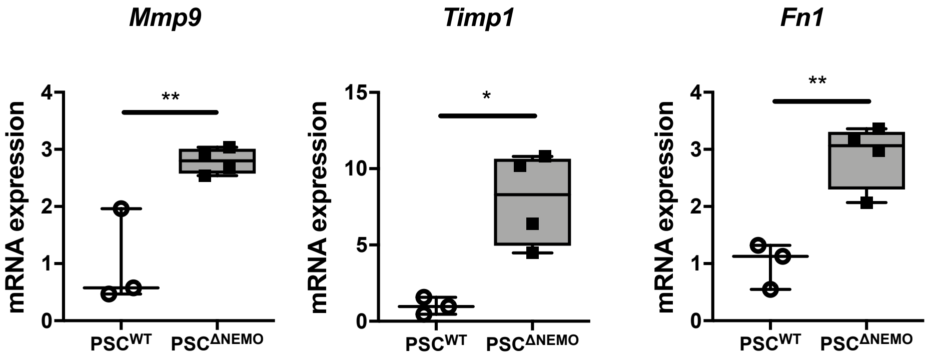

Supplementary figure 8. PSC$^{\Delta NEMO}$ cells show upregulation of ECM components. Expressions of genes involved in ECM modifications in PSCs analyzed by qPCR (n≥3). Whiskers: Min to Max. T-test (two-tailed): *p<0.05; **p<0.01. The n number represents biological replicates.

Supplementary figure 9. Original Western blot images

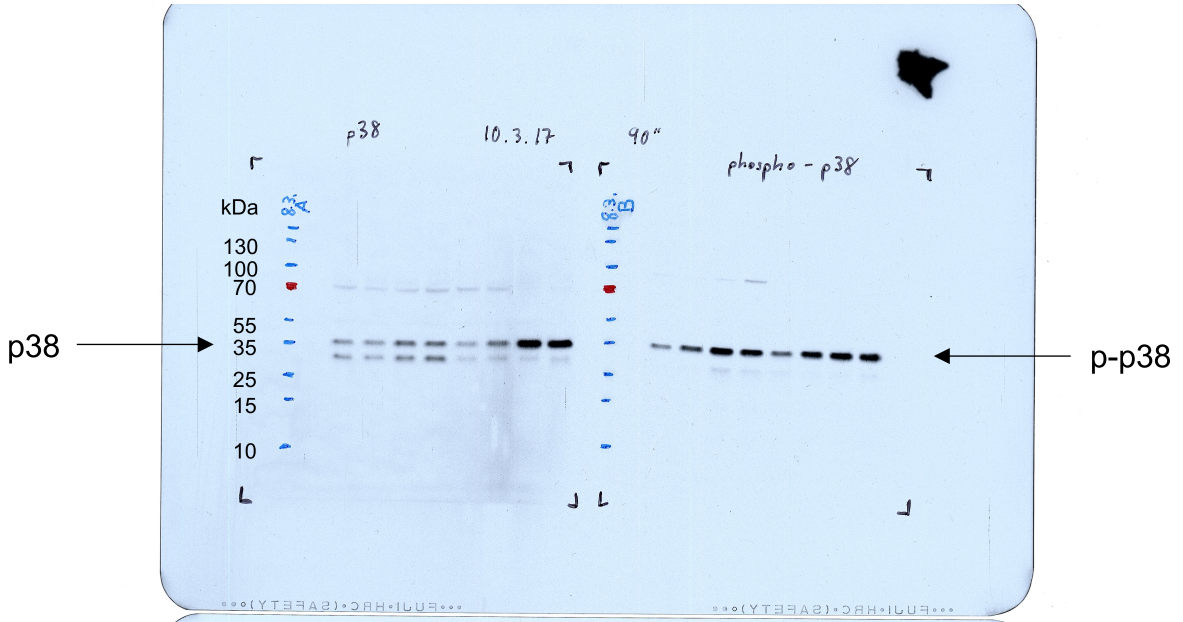

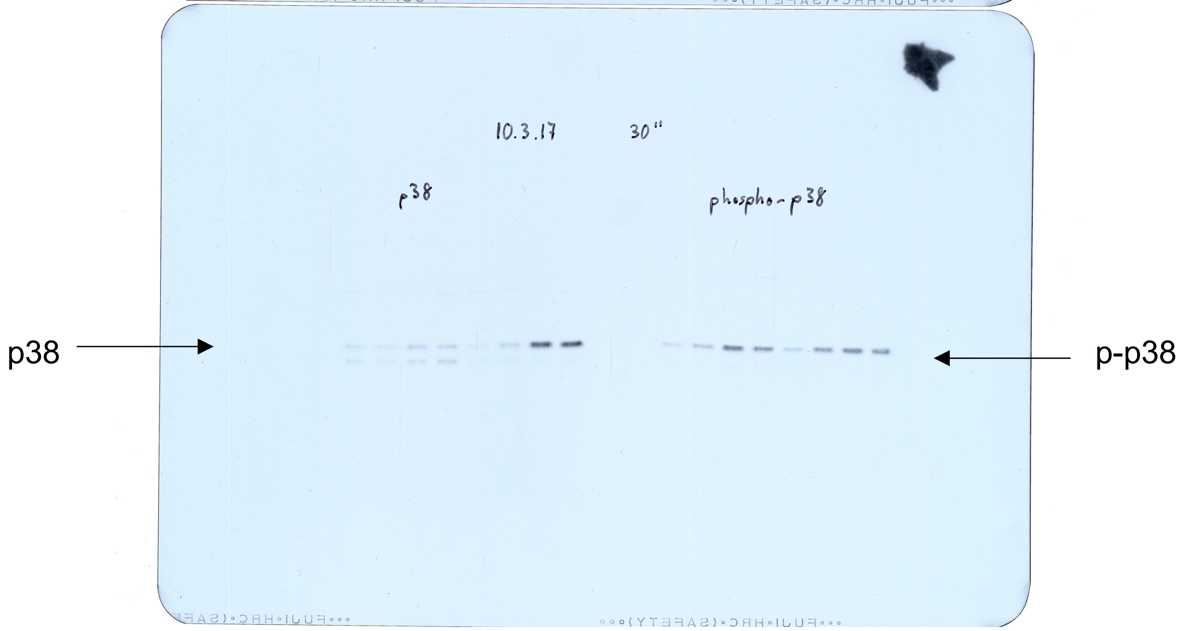

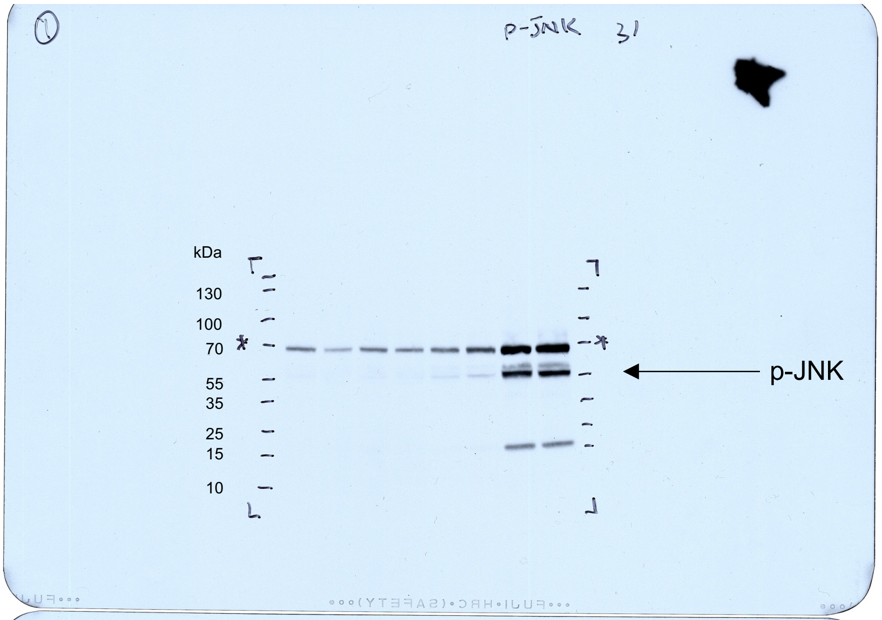

p-JNK 31
kDa
130
100
70
55
35
25
15
10
p-JNK

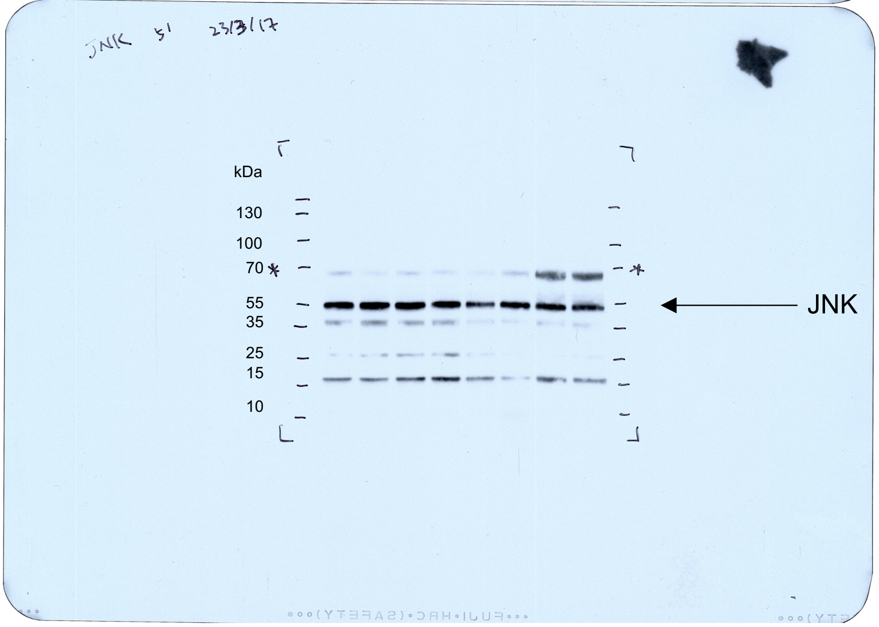

JNK 5'
23/3/17
kDa
130
100
70
55
35
25
15
10
JNK

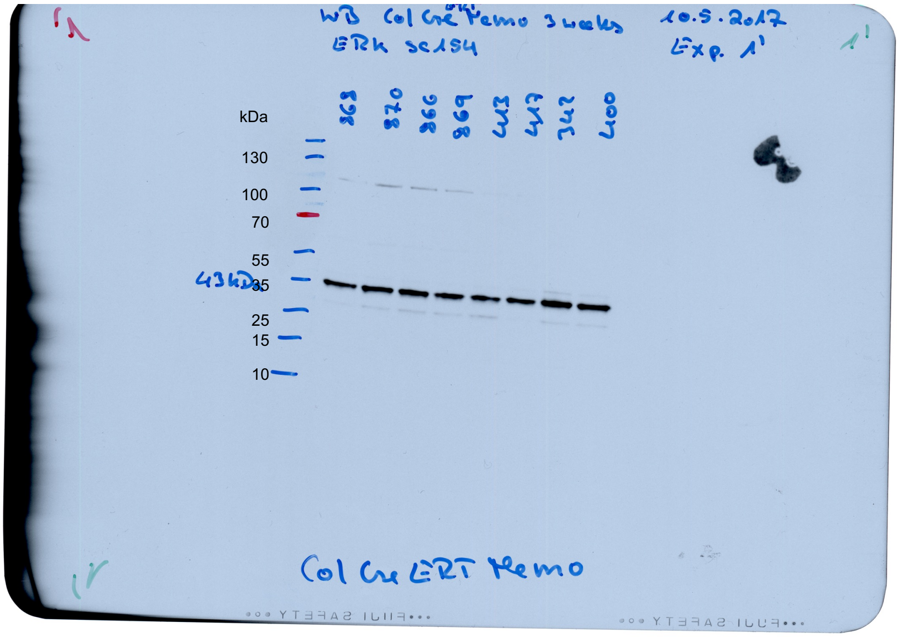
kDa
130
100
70
55
35
25
15
10

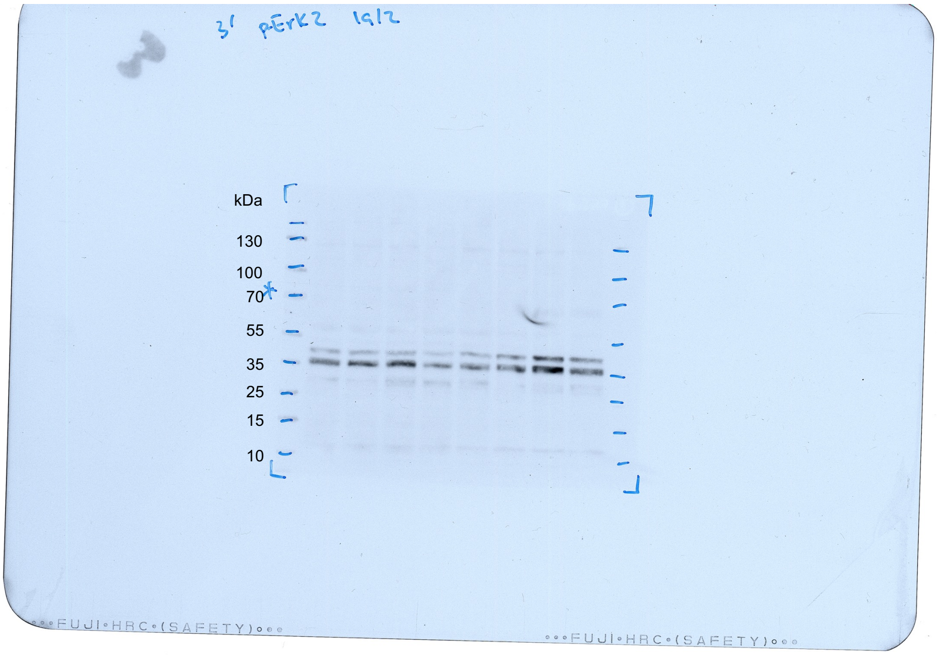
kDa
130
100
70
55
35
25
15
10

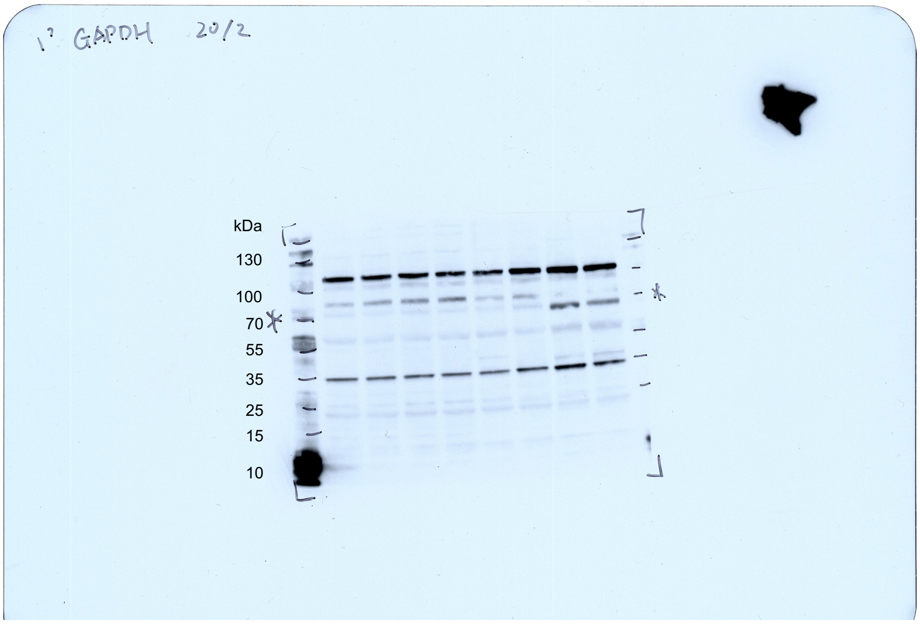
kDa
130
100
70
55
35
25
15
10

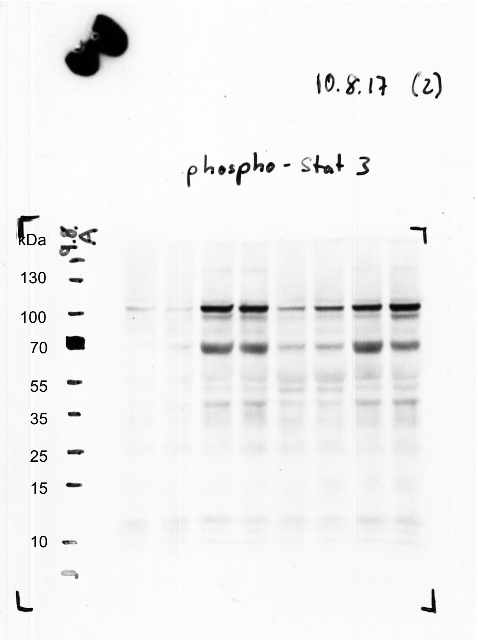
10.8.17 (2)
phospho - Stat 3
kDa
130
100
70
55
35
25
15
10

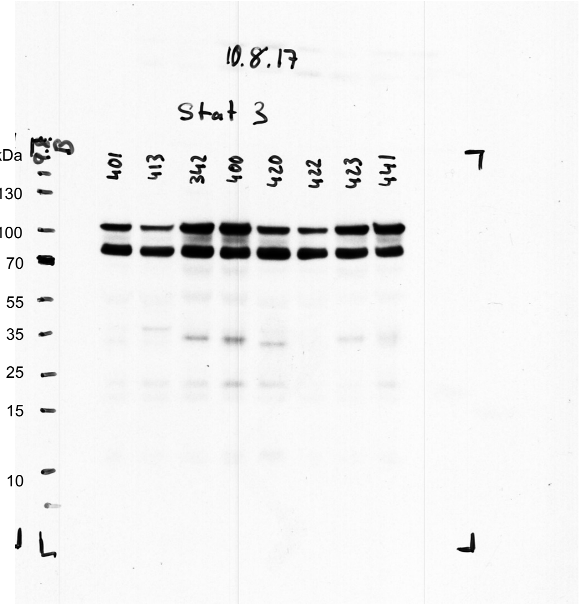
10.8.17
Stat 3
kDa
401
413
342
400
420
422
423
441
130
100
70
55
35
25
15
10

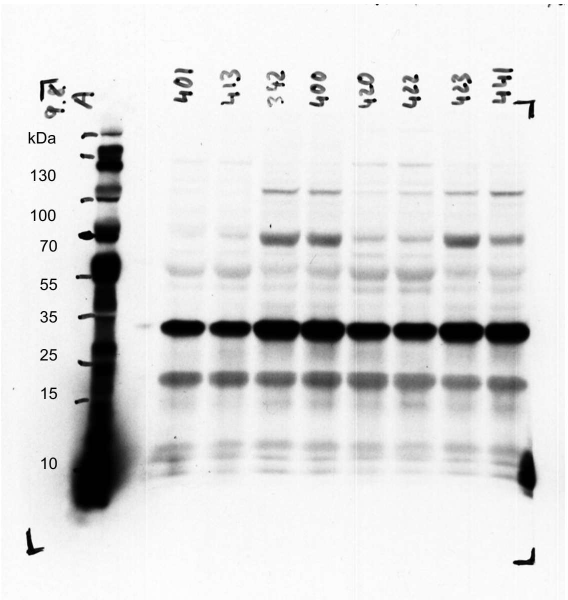
401
413
342
400
420
422
423
441
kDa
130
100
70
55
35
25
15
10

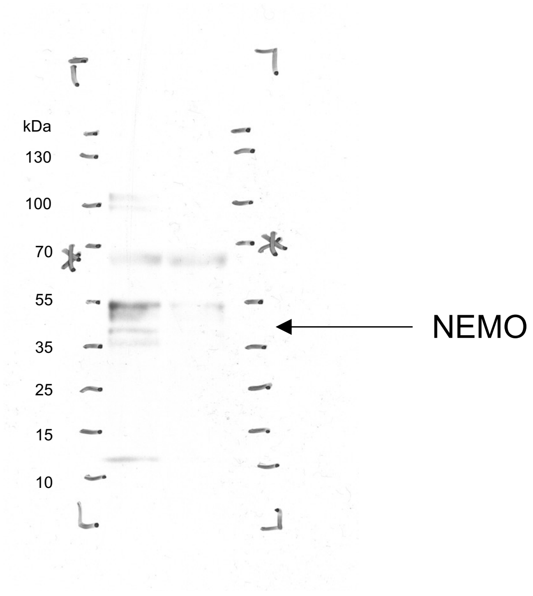
kDa
130
100
70
55
35
25
15
10
NEMO

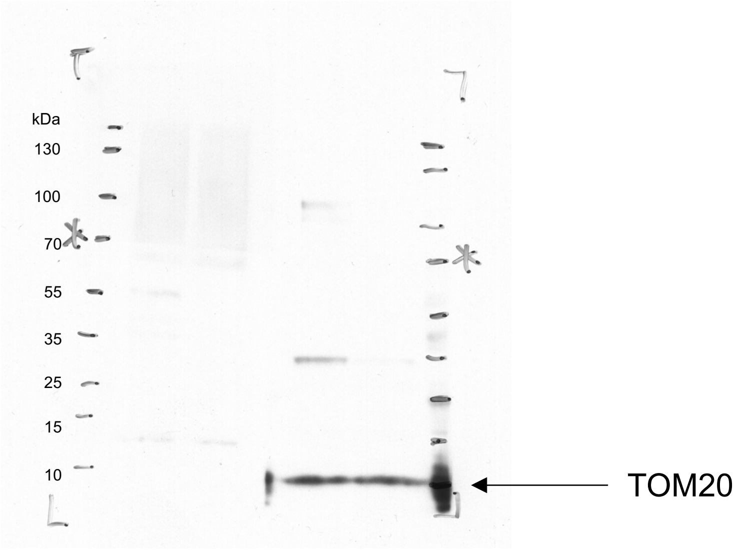
kDa
130
100
70
55
35
25
15
10
TOM20

Supplementary Tables

**Primary antibody**	**Dilutions**	**Company**
rabbit anti-α-amylase	1:400	Sigma (A8273)
goat anti-CK19	1:100	Santa Cruz (sc-33111)
rat anti-CD45	1:100	BD Pharmingen (#550539)
mouse anti-αSMA	1:100	Millipore (CBL171)
rabbit anti-RFP	1:100	Rockland (600-401-379)
rat anti-B220	1:100	BioLegend (103247)
rat anti-CD3	1:100	BioLegend (100201)
rat anti-CD4	1:100	BD Pharmingen (550278)
rat anti-CD8	1:100	BD Pharmingen (550281)
rabbit anti-vimentin	1:100	abcam (ab7783)
rat anti-SiglecF	1:100	Miltenyi Biotec (130-102-167)
goat anti-nestin	1:100	Santa Cruz (sc-21248)

Supplementary table 1. List of primary antibodies for histological staining.

**Antibody**	**Dilutions**	**Company**
CD11c (BV510)	1:100	BioLegend (117337)
CD11b (APC)	1:100	BioLegend (101212)
CD45 (APC/Cy7)	1:400	BD Pharmingen (557659)
Ly6G (BV421)	1:100	BioLegend (127627)
CCR3 (PerCP)	1:100	R&D System (FAB729C-025)
SiglecF (PE-Vio770)	1:25	MACS (130-102-167)
CD4 (FITC)	1:100	MACS (120-001-955)
CD4 (PE)	1:100	BioLegend (100407)
IL17A (APC)	1:50	MACS (130-103-027)
F4/80 (AF448)	1:50	Bio-Rad (MCA497A448T)
CD3ε (PE/Cy7)	1:100	BioLegend (100320)

Supplementary table 2. List of antibodies for flow cytometry.

**Primary antibodies**	**Dilutions**	**Company**
NEMO	1:1000	BD Transduction Laboratories (#611306)
GAPDH	1:1000	Santa Cruz (sc-25778)
TOM20	1:1000	Santa Cruz (sc-11415)
p-JNK	1:1000	Cell Signaling (9255S)
JNK	1:1000	Santa Cruz (sc-7345)
p-p38	1:1000	Cell Signaling (9211S)
p38	1:1000	Cell Signaling (9212)
p-ERK	1:1000	Cell Signaling (4377)
ERK2	1:1000	Santa Cruz (sc-154)

p-STAT3	1:1000	Cell Signaling (9145S)
STAT3	1:1000	Cell Signaling (4904S)

Supplementary table 3. List of antibodies for western blot.

Gene	Primer Sequence	Gene	Primer Sequence
Col1α1	F 5’ catgttcagctttgtggacct 3’<br>R 5’ gcagctgacttcagggatgt 3’	***Icam1***	F 5’ cccacgctacctctgctc 3’<br>R 5’ gatggatacctgagcatcacc 3’
Col1α2	F 5’ agggcctgatggaaacaag 3’<br>R 5’ cccctctctcctggaagc 3’	***Nfkbia***	F 5’ cagaacaacctgcagcagac 3’<br>R 5’ tttcagaagtgcctcagcaat 3’
Col3α1	F 5’ tcccctggaatctgtgaatc 3’<br>R 5’ tgagtcgaattggggagaat 3’	***Ccl6***	F 5’ tctttatccttgtggctgtcc 3’<br>R 5’ tggagggttatagcgacgat 3’
Vim	F 5’ gtaccggagacaggtgcagt 3’<br>R 5’ ttctcttccatctcacgcatc 3’	***Ccl8***	F 5’ tcagcccagagaagctgac 3’<br>R 5’ gggggatcttcagctttagtaca 3’
Cxcr4	F 5’ tggaaccgatcagtgtgagt 3’<br>R 5’ gggcaggaagatcctattga 3’	***Ccl9***	F 5’ ggctgctcaggtttcactgt 3’<br>R 5’ agaacgatagggcggtaggt 3’
Fn1	F 5’ gatgccgatcagaagtttgg 3’<br>R 5’ ggttgtgcagatctcctcgt 3’	***Hprt***	F 5’ ggagcggtagcacctcct 3’<br>R 5’ cctggttcatcatcgctaatc 3’
Tgfb1	F 5’ tggagcaacatgtggaactc 3’<br>R 5’ gtcagcagccggttacca 3’	***Ccl11***	F 5’ agagctccacagcgcttct 3’<br>R 5’ gcaggaagttgggatgga 3’
Rpl13	F 5’ cctgctgctctcaaggttgt 3’<br>R 5’ ggtacttccacccgacctc 3’	***Ccl12***	F 5’ ccatcagtcctcaggtattgg 3’<br>R 5’ cttccggacgtgaatcttct 3’
Il6	F 5’ gctaccaaactggatataatcagga 3’<br>R 5’ ccaggtagctatggtactccagaa 3’	***Ccl24***	F 5’ gtgcctgacctccagaacat 3’<br>R 5’ gaggggatggtcacagaatc 3’
Prg2	F 5’ ggagcgtctgctcttcatct 3’<br>R 5’ cccctggaggacactcttct 3’	***Ccr2***	F 5’ acctgtaaatgccatgcaagt 3’<br>R 5’ tgtcttccatttcctttgatttg 3’
Ifng	F 5’ atctggaggaactggcaaaa3’<br>R 5’ ttcaagacttcaaagagtctgagg 3’	***Ccr3***	F 5’ gaatcaaagagctggggtca 3’<br>R 5’ caggaggccgatgatgaa 3’
Il12a	F 5’ ccaggtgtcttagccagtcc 3’<br>R 5’ gcagtgcaggaataatgtttca 3’	***Siglecf***	F 5’ gaacttacctggcactggtgtt 3’<br>R 5’ gctccactctgcaggacttt 3’
Il4	F 5’ cctgctcttctttctcgaatgt 3’<br>R 5’ cacatccatctccgtgcat 3’	***Ikbkg***	F 5’ ctggcttcaagtcacccaag 3’<br>R 5’ gtgctaaagcaccacccagt 3’
Il5	F 5’ acattgaccgccaaaaagag 3’<br>R 5’ atccaggaactgcctcgtc 3’	***Cpa1***	F 5’ agcagtcttcggcaatgag 3’<br>R 5’ cagaagtccaacttcaagtgctc 3’
Il17	F 5’ ggacagcccttctttgtctg 3’<br>R 5’ tgctttttatatttcattacgtggtt 3’		

Supplementary table 4. List of primers for qPCR.
