# Supplementary material for: Functional IKK/NF-κB signaling in pancreatic stellate cells is essential to prevent autoimmune pancreatitis
Source: Commun Biol. 2022 May 27;5:509. doi: 10.1038/s42003-022-03371-3 (PMC9142538; doi:10.1038/s42003-022-03371-3)
Supplement: Supplementary file 3 — Description of Additional Supplementary Files [file 42003_2022_3371_MOESM3_ESM.pdf]

Description of Additional Supplementary Files

File name: Supplementary Data 1
Description: All raw data used in plotting graphs.
