# Supplementary material for: Functional IKK/NF-κB signaling in pancreatic stellate cells is essential to prevent autoimmune pancreatitis
Source: Commun Biol. 2022 May 27;5:509. doi: 10.1038/s42003-022-03371-3 (PMC9142538; doi:10.1038/s42003-022-03371-3)
Supplement: Supplementary file 5 — Reporting Summary [file 42003_2022_3371_MOESM5_ESM.pdf]

nature portfolio

Corresponding author(s): Prof. Thomas Wirth

Last updated by author(s): Mar 22, 2022

Reporting Summary

Nature Portfolio wishes to improve the reproducibility of the work that we publish. This form provides structure for consistency and transparency in reporting. For further information on Nature Portfolio policies, see our Editorial Policies and the Editorial Policy Checklist.

Statistics

For all statistical analyses, confirm that the following items are present in the figure legend, table legend, main text, or Methods section.

n/a	Confirmed	
☐	☒	The exact sample size (n) for each experimental group/condition, given as a discrete number and unit of measurement
☐	☒	A statement on whether measurements were taken from distinct samples or whether the same sample was measured repeatedly
☐	☒	The statistical test(s) used AND whether they are one- or two-sided<br>*Only common tests should be described solely by name; describe more complex techniques in the Methods section.*
☒	☐	A description of all covariates tested
☐	☒	A description of any assumptions or corrections, such as tests of normality and adjustment for multiple comparisons
☐	☒	A full description of the statistical parameters including central tendency (e.g. means) or other basic estimates (e.g. regression coefficient) AND variation (e.g. standard deviation) or associated estimates of uncertainty (e.g. confidence intervals)
☒	☐	For null hypothesis testing, the test statistic (e.g. F, t, r) with confidence intervals, effect sizes, degrees of freedom and P value noted<br>*Give P values as exact values whenever suitable.*
☒	☐	For Bayesian analysis, information on the choice of priors and Markov chain Monte Carlo settings
☒	☐	For hierarchical and complex designs, identification of the appropriate level for tests and full reporting of outcomes
☒	☐	Estimates of effect sizes (e.g. Cohen's d, Pearson's r), indicating how they were calculated

Our web collection on statistics for biologists contains articles on many of the points above.

Software and code

Policy information about availability of computer code

Data collection	Not applicable.
Data analysis	Not applicable.

For manuscripts utilizing custom algorithms or software that are central to the research but not yet described in published literature, software must be made available to editors and reviewers. We strongly encourage code deposition in a community repository (e.g. GitHub). See the Nature Portfolio guidelines for submitting code & software for further information.

Data

Policy information about availability of data

All manuscripts must include a data availability statement. This statement should provide the following information, where applicable:

- Accession codes, unique identifiers, or web links for publicly available datasets
- A description of any restrictions on data availability
- For clinical datasets or third party data, please ensure that the statement adheres to our policy

The microarray data was deposited in ArrayExpress with accession number E-MTAB-7274.

Field-specific reporting

Please select the one below that is the best fit for your research. If you are not sure, read the appropriate sections before making your selection.

☒ Life sciences ☐ Behavioural & social sciences ☐ Ecological, evolutionary & environmental sciences

For a reference copy of the document with all sections, see nature.com/documents/nr-reporting-summary-flat.pdf

Life sciences study design

All studies must disclose on these points even when the disclosure is negative.

Sample size	For animal experiments, we used a minimum sample size of 4. From our previous studies using the caerulein-induced pancreatitis model, we found that a sample size between 4-8 was enough to see a statistical significance in our experiments.
Data exclusions	No data was excluded.
Replication	We used biological replicates for all animal experiments.
Randomization	We used block randomization to ensure both genders fell into experimental and control groups.
Blinding	Investigators were blinded when applicable. e.g. Acquisition of microscopic pictures for image analyses and quantifications.

Reporting for specific materials, systems and methods

We require information from authors about some types of materials, experimental systems and methods used in many studies. Here, indicate whether each material, system or method listed is relevant to your study. If you are not sure if a list item applies to your research, read the appropriate section before selecting a response.

Materials & experimental systems

n/a	Involved in the study	
☐	☒	Antibodies
☐	☒	Eukaryotic cell lines
☒	☐	Palaeontology and archaeology
☐	☒	Animals and other organisms
☒	☐	Human research participants
☒	☐	Clinical data
☒	☐	Dual use research of concern

Methods

n/a	Involved in the study	
☒	☐	ChIP-seq
☐	☒	Flow cytometry
☒	☐	MRI-based neuroimaging

Antibodies

Antibodies used

CD11c (BV510) BioLegend (117337)
CD11b (APC) BioLegend (101212)
CD45 (APC/Cy7) BD Pharmingen (557659)
Ly6G (BV421) BioLegend (127627)
CCR3 (PerCP) R&D System (FAB729C-025)
SiglecF (PE-Vio770) MACS (130-102-167)
CD4 (FITC) MACS (120-001-955)
CD4 (PE) BioLegend (100407)
IL17A (APC) MACS (130-103-027)
Col1a1 Rockland (600-406-103)
F4/80 (AF448) Bio-Rad (MCA497A448T)
CD3ε (PE/Cy7) BioLegend (100320)
NEMO BD Transduction Laboratories (#611306)
GAPDH Santa Cruz (sc-25778)
TOM20 Santa Cruz (sc-11415)
p-JNK Cell Signaling (9255S)
JNK Santa Cruz (sc-7345)
p-p38 Cell Signaling (9211S)
p38 Cell Signaling (9212)
p-ERK Cell Signaling (4377)
ERK2 Santa Cruz (sc-154)
p-STAT3 Cell Signaling (9145S)
STAT3 Cell Signaling (4904S)
rabbit anti-α-amylase Sigma (A8273)

goat anti-CK19 Santa Cruz (sc-33111)
rat anti-CD45 BD Pharmingen (#550539)
mouse anti-αSMA Millipore (CBL171)
rabbit anti-RFP Rockland (600-401-379)
rat anti-B220 BioLegend (103247)
rat anti-CD3 BioLegend (100201)
rat anti-CD4 BD Pharmingen (550278)
rat anti-CD8 BD Pharmingen (550281)
rabbit anti-vimentin abcam (ab7783)
rat anti-SiglecF Miltenyi Biotec (130-102-167)
goat anti-nestin Santa Cruz (sc-21248)
rabbit anti-CCR2 abcam (ab32144)

Validation: All the antibodies were validated by the manufacturers with the data provided in their corresponding datasheet.

Eukaryotic cell lines

Policy information about cell lines

Cell line source(s)	Primary pancreatic stellate cell lines derived mice used in this study.
Authentication	Stainings of several markers were performed to verify their cell identity.
Mycoplasma contamination	The cell lines were not checked for mycoplasma contamination.
Commonly misidentified lines (See ICLAC register)	Not applicable.

Animals and other organisms

Policy information about studies involving animals; ARRIVE guidelines recommended for reporting animal research

Laboratory animals	All mice used in this study were in C57BL/6 background. Both genders were used in all experiments.
Wild animals	Not applicable.
Field-collected samples	Not applicable.
Ethics oversight	All animal experiments followed the relevant ethical regulations and were performed in accordance to the German animal welfare legislation with approvals from the responsible organization.

Note that full information on the approval of the study protocol must also be provided in the manuscript.

Flow Cytometry

Plots

Confirm that:

☒ The axis labels state the marker and fluorochrome used (e.g. CD4-FITC).

☒ The axis scales are clearly visible. Include numbers along axes only for bottom left plot of group (a 'group' is an analysis of identical markers).

☒ All plots are contour plots with outliers or pseudocolor plots.

☒ A numerical value for number of cells or percentage (with statistics) is provided.

Methodology

Sample preparation	Immune cells were isolated from mice from the tissue and the blood circulation. For the isolation of immune cells from tissue, the tissue was digested with collagenase D before performing a gradient centrifugation using Histopaque 1077 and 1119. The cells were used directly for flow cytometry. For the isolation of immune cells from blood circulation, whole blood was collected from the mice upon euthanization and treated with RBC lysis buffer before using in flow cytometry.
Instrument	BD CantoII
Software	BD FACSDiva
Cell population abundance	CD45+CD11b+CD11c-Ly6G-CCR3+Siglec-F+ eosinophil population was identified in our target mice to be 23.7% and 7.5% of the total CD45+ cells isolated from the tissue and blood circulation respectively.

Gating strategy	We first used FSC-A vs. SSC-A to identify all cells, then used FSC-A vs. FSC-H to gate for single cells. For gating the viable cells, we only took the sytox negative population. We then focused all the analyses on the CD45+ populations.

☒ Tick this box to confirm that a figure exemplifying the gating strategy is provided in the Supplementary Information.
